# Association between early treatment response and long-term smoking cessation: A real-world retrospective cohort study of cytisine treatment

**DOI:** 10.18332/tid/241540

**Published:** 2026-11-05

**Authors:** Yakup Arslan, Burcu Öztürk Şahin, Arzu Aygül, Cansu Yaman, Zeynep Aytül Selimoğlu, Gökberk Sızı, Eljan Heydarov, Ebru Öztürk, Esra Sönmez, Deniz Doğan, Nesrin Öcal, Cantürk Taşçı

**Affiliations:** 1 Gülhane Faculty of Medicine, Department of Pulmonary Medicine, Health Sciences UniversityAnkaraTürkiye; 2 Gülhane Faculty of Medicine, Health Sciences UniversityAnkaraTürkiye; 3 Faculty of Medicine, Department of Biostatistics, Hacettepe UniversityAnkaraTürkiye

**Keywords:** cytisine treatment, early treatment response, long-term smoking cessation

## Abstract

**Introduction:**

Cytisine is an effective and cost-efficient pharmacotherapy for smoking cessation; however, real-world evidence regarding treatment outcomes within structured behavioral programs remains limited. This study aimed to evaluate short- and mid-term smoking cessation outcomes following cytisine treatment and to identify factors associated with treatment success, with particular emphasis on early treatment response as a marker of long-term abstinence.

**Methods:**

This retrospective cohort study included 364 consecutive adult smokers who initiated cytisine treatment within a structured smoking cessation program at a tertiary outpatient clinic between April 2023 and February 2025. Smoking abstinence was assessed at 1 and 6 months through outpatient visits and structured telephone interviews recorded in the Turkish National Smoking Cessation Registry System (TUBATIS). Baseline demographic characteristics, nicotine dependence assessed using the Fagerström test for nicotine dependence (FTND), cumulative smoking exposure, and previous smoking cessation treatment were evaluated. Multivariable logistic regression, interaction analyses, receiver operating characteristic (ROC) curve analyses, and Kaplan–Meier survival analyses were performed.

**Results:**

Smoking abstinence was achieved by 114 patients (31.3%) at 1 month and 67 (18.4%) at 6 months. Early responders had substantially higher 6-month abstinence rates than early non-responders (49.1% vs 4.4%), and early treatment response was strongly associated with sustained abstinence after multivariable adjustment (AOR=9.8; 95% CI: 4.7–20.5; p<0.001). Lower cumulative smoking exposure (≤20 pack-years) was independently associated with early abstinence (AOR=3.55; 95% CI: 1.99–6.36; p<0.001), whereas sex, nicotine dependence, age, and previous cessation treatment were not independently associated with 1-month abstinence. A significant interaction between nicotine dependence and smoking exposure was observed (p=0.028). A prediction model incorporating early treatment response demonstrated better discrimination for 6-month abstinence than a baseline clinical model (AUC: 0.82 vs 0.68).

**Conclusions:**

Early treatment response was strongly associated with sustained smoking abstinence and may represent a clinically useful time point for treatment reassessment. Baseline nicotine dependence and cumulative smoking exposure were also associated with cessation outcomes, supporting individualized risk assessment and follow-up. Cytisine, when combined with structured behavioral support, was associated with clinically meaningful smoking cessation outcomes in this real-world cohort.

## Introduction

Tobacco smoking remains one of the leading preventable causes of morbidity and mortality worldwide, accounting for more than 8 million deaths annually and imposing a substantial burden on healthcare systems (ICD-10 F17)^1^. Despite the well-established health risks, tobacco dependence remains highly prevalent because nicotine addiction is a chronic relapsing condition driven by complex biological, psychological, and behavioral mechanisms. Nicotine exerts its addictive effects primarily through activation of α4β2 nicotinic acetylcholine receptors^2^. Therefore, effective smoking cessation requires an integrated approach combining behavioral interventions with evidence-based pharmacotherapy.

Cytisine, a plant-derived alkaloid obtained from *Laburnum anagyroides*, is a partial agonist of α4β2 nicotinic receptors with a pharmacological profile similar to varenicline. By partially stimulating these receptors while simultaneously blocking nicotine binding, cytisine reduces withdrawal symptoms and attenuates the rewarding effects of smoking^2,3^.

Over the past decades, randomized trials and meta-analyses have demonstrated the efficacy and safety of cytisine, with cessation rates comparable to established pharmacotherapies^4^. In addition to its clinical effectiveness, cytisine offers important advantages including low cost, short treatment duration, and favorable tolerability, making it particularly suitable for large-scale public health implementation^4-6^.

Despite increasing global use, real-world data evaluating cytisine within structured cessation programs remain limited. Moreover, traditional models of smoking cessation focus predominantly on baseline characteristics, while the role of early treatment response as a dynamic predictor of long-term outcomes has not been sufficiently explored^6^.

Unlike previous real-world studies that have primarily focused on baseline patient characteristics, the present study evaluates early treatment response as a dynamic clinical marker associated with long-term smoking cessation. By integrating early clinical response into predictive modeling, this study aims to provide clinically relevant evidence to support individualized follow-up and treatment optimization in routine smoking cessation practice.

The present retrospective cohort study aimed to evaluate demographic and clinical factors associated with smoking cessation outcomes among adults receiving cytisine treatment in a real-world smoking cessation outpatient clinic and to assess the relationship between early treatment response and sustained abstinence at 6 months.

## Methods

### Study design and population

This retrospective observational cohort study was conducted at the Smoking Cessation Outpatient Clinic of Gülhane Training and Research Hospital and included consecutive adult smokers who initiated cytisine treatment between April 2023 and February 2025. All eligible patients aged ≥18 years who received cytisine as part of the structured smoking cessation program were included in the study cohort. The source cohort was defined *a priori* as all consecutive adult patients who initiated cytisine during the study period; therefore, no broader pharmacotherapy-treated population was screened for eligibility.

The study was approved by the Scientific Research Ethics Committee of Gülhane Faculty of Medicine, University of Health Sciences (Decision No. 2025–161; 24 June 2025). Owing to the retrospective study design, the requirement for written informed consent was waived. All procedures were conducted in accordance with the Declaration of Helsinki.

### Smoking cessation program

All participants received a standardized smoking cessation program consisting of cytisine pharmacotherapy combined with motivational interviewing (MI) and cognitive behavioral therapy (CBT). Smoking status was routinely assessed at 1 and 6 months through outpatient visits and structured telephone interviews, with all follow-up data recorded in the Turkish National Smoking Cessation Registry System (TUBATIS).

A total of 364 consecutive patients initiated cytisine treatment and constituted the study cohort. During follow-up, 238 patients (65.4%) completed all scheduled follow-up assessments, whereas 126 patients (34.6%) did not complete scheduled follow-up. Among the 126 participants who did not complete scheduled follow-up visits, smoking status was ascertained from available electronic health records and telephone contact, confirming that they had not achieved smoking abstinence. As all had initiated cytisine treatment, they were retained in the study cohort and classified as treatment failures in the statistical analyses. Follow-up completion was recorded descriptively. The primary analyses were performed using the entire study cohort. Smoking abstinence was defined as self-reported complete abstinence from smoking at the respective follow-up assessment (Figure 1).

**Figure 1:**
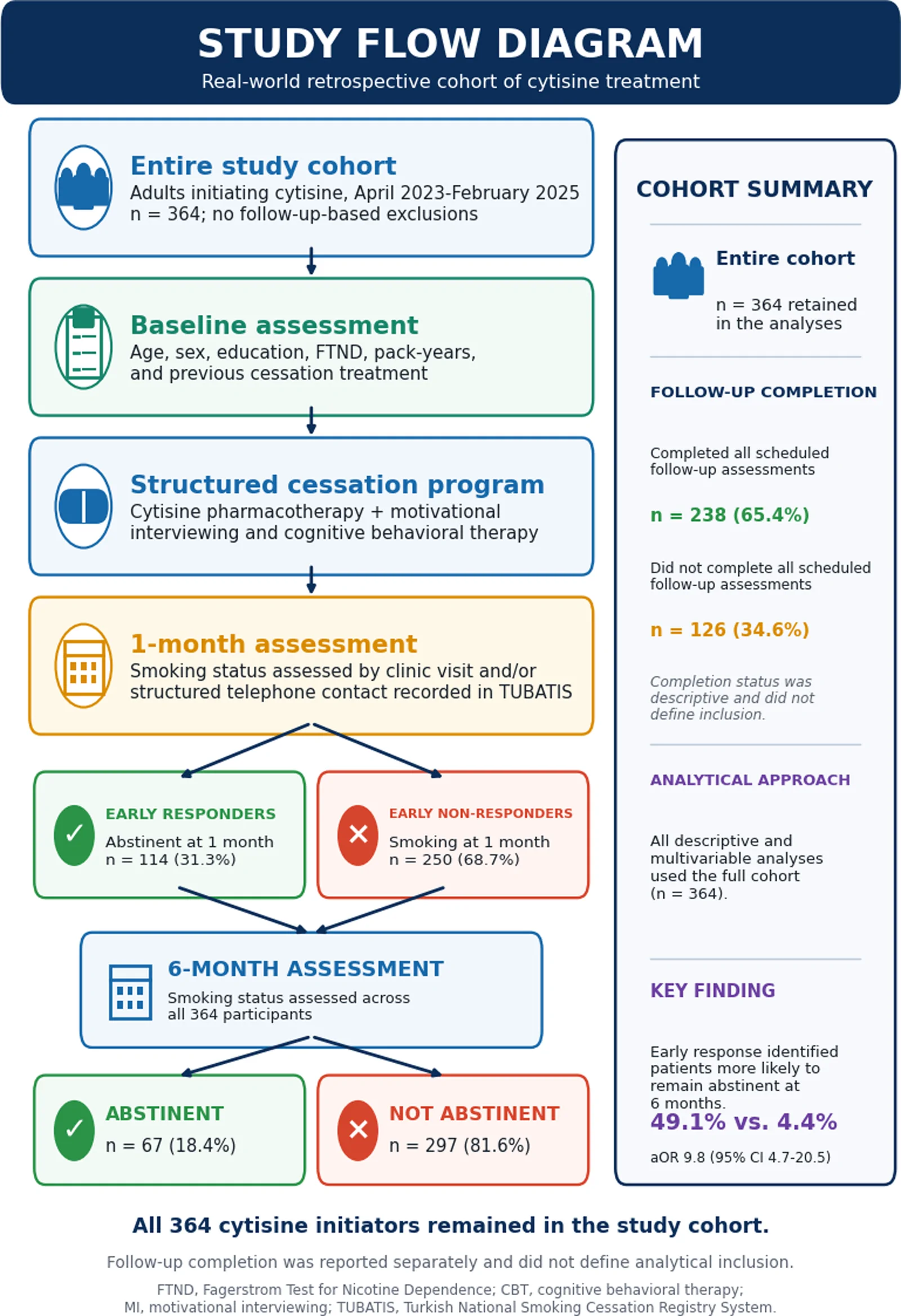
Study flow diagram

All 364 consecutive adults who initiated cytisine between April 2023 and February 2025 constituted the study cohort and were retained in the analyses. Smoking status was assessed at 1 and 6 months through outpatient visits and structured telephone contact recorded in TUBATIS. Follow-up completion was reported descriptively and did not determine analytical inclusion. Early responders reported abstinence at 1 month. CBT: cognitive behavioral therapy. MI: motivational interviewing. FTND: Fagerström test for nicotine dependence.

### Variables and outcomes

Baseline demographic and clinical variables included age, sex, education level, body mass index, smoking duration, cumulative smoking exposure (pack-years), previous smoking cessation treatment, and nicotine dependence assessed using the Fagerström test for nicotine dependence (FTND)^7^. FTND scores were categorized as low (≤3), moderate (4–6), or high (≥7). Smoking exposure was categorized as <10, 10–20, and >20 pack-years.

The exposure of interest was treatment with cytisine administered within the structured smoking cessation program. Early treatment response was defined as self-reported smoking abstinence at the 1-month follow-up. Participants who achieved abstinence at 1 month were classified as early responders, whereas those who continued smoking were classified as early non-responders.

The primary outcome was sustained smoking abstinence at 6 months. Smoking status at both 1 and 6 months was determined from outpatient assessments and structured telephone interviews documented in TUBATIS. Participants reporting complete abstinence at the respective follow-up assessments were classified as abstinent. The 1-month model was parameterized for smoking abstinence to identify factors associated with early treatment success, whereas the 6-month model was parameterized for failure to achieve abstinence to identify baseline characteristics associated with long-term treatment failure. Accordingly, odds ratios >1 represent higher odds of success in the 1-month model and higher odds of failure in the 6-month model.

### Potential covariates

Potential covariates included age, sex, education level, body mass index, smoking duration, cumulative smoking exposure (pack-years), FTND score, number of previous quit attempts, and smoking-related comorbidities. Variables considered clinically relevant based on previous literature, together with variables associated with the outcome in univariable analyses, were entered into the multivariable logistic regression models.

### Statistical analysis

Continuous variables were summarized as mean ± standard deviation or median (interquartile range), as appropriate according to data distribution. Categorical variables were expressed as frequencies and percentages. Normality was assessed using the Shapiro–Wilk test together with histogram, Q–Q plot, and boxplot inspection. Comparisons between groups were performed using the Kruskal–Wallis test for continuous variables and the χ² test or Fisher’s exact test for categorical variables, as appropriate. Spearman’s rank correlation coefficient was used to assess correlations between continuous variables. Participants who did not complete scheduled follow-up assessments but were confirmed not to have achieved smoking abstinence were classified as treatment failures and retained in the full cohort for regression, ROC, and survival analyses; no outcome imputation was performed.

Predictors of smoking cessation were evaluated using multivariable logistic regression analyses. Interaction analyses were performed to investigate the joint effects of nicotine dependence and cumulative smoking exposure. Model discrimination was assessed using receiver operating characteristic (ROC) curve analysis and quantified by the area under the curve (AUC). Time to relapse was evaluated using Kaplan–Meier survival analysis with comparisons performed using the log-rank test. Two-sided p<0.05 was considered statistically significant. All analyses were performed using IBM SPSS Statistics version 23 (IBM Corp., Armonk, NY, USA).

## Results

### Baseline characteristics

A total of 364 consecutive patients who initiated cytisine treatment were included in the study cohort (Figure 1). During follow-up, 238 patients (65.4%) completed all scheduled follow-up assessments, whereas 126 patients (34.6%) did not complete scheduled follow-up.

The mean age of the study population was 46.1 ± 12.9 years, with a median age of 48 years (IQR: 37–56). Women accounted for 51.1% (n=186) of the cohort and men for 48.9% (n=178). Most participants had completed high school (48.1%), followed by university education (37.9%), whereas 14.0% had primary school education. The median cumulative smoking exposure was 25 pack-years (IQR: 15–40), and 66.5% of participants had smoked more than 20 pack-years. Nicotine dependence was predominantly high, with 278 patients (76.4%) classified as having high FTND scores, while 47 (12.9%) and 39 (10.7%) had moderate and low nicotine dependence, respectively. Previous smoking cessation treatment had been received by 132 patients (36.3%), whereas 232 (63.7%) were treatment-naïve (Table 1).

**Table 1:** Baseline characteristics and comparison according to 1-month smoking abstinence status

Characteristics	Overall cohort (N=364) n (%)	Early non-responders (N=250) n (%)	Early responders (N=114) n (%)	p
**Age** (years), mean ± SD	46.1 ± 12.9	47.5 ± 10.9	44.1 ± 13.3	0.010
**Sex** (n, %)				
Male	178 (48.9)	112 (44.8)	66 (57.9)	0.001
Female	186 (51.1)	138 (55.2)	48 (42.1)	
**Education level**				
Primary school	51 (14.0)	42 (16.8)	9 (7.9)	0.065
High school	175 (48.1)	114 (45.6)	61 (53.5)	
Higher education	138 (37.9)	94 (37.6)	44 (38.6)	
**FTND category**				
Low (≤3)	39 (10.7)	23 (9.2)	16 (14.0)	<0.001
Moderate (4–6)	47 (12.9)	24 (9.6)	23 (20.2)	
High (≥7)	278 (76.4)	203 (81.2)	75 (65.8)	
**Cumulative smoking exposure** (pack-years)				
<10	66 (18.1)	34 (13.6)	32 (28.1)	0.004
10–20	56 (15.4)	39 (15.6)	17 (14.9)	
>20	242 (66.5)	177 (70.8)	65 (57.0)	
**Previous smoking-cessation treatment**				
Treatment-naïve	232 (63.7)	150 (60.0)	82 (71.9)	**0.018**
Previously treated	132 (36.3)	100 (40.0)	32 (28.1)	
**Previous treatment**				
Nicotine replacement therapy	44 (12.1)	29 (11.6)	15 (13.2)	0.672
Q-smoke	79 (21.7)	50 (20.0)	29 (25.4)	0.243
Varenicline (Champix)	62 (17.0)	49 (19.6)	13 (11.4)	0.054

FTND: Fagerström test for nicotine dependence.

### Smoking cessation outcomes

Smoking abstinence was achieved by 114 patients (31.3%) at the 1-month assessment and by 67 patients (18.4%) at 6 months. Conversely, 250 patients (68.7%) continued smoking at 1 month, and 297 (81.6%) had either failed to quit or relapsed by the follow-up at 6 months.

### Predictors of early smoking abstinence

In univariable analyses, younger age, male sex, lower nicotine dependence, lower cumulative smoking exposure, and the absence of previous smoking cessation treatment were associated with successful smoking cessation at 1 month (Figure 2).

**Figure 2:**
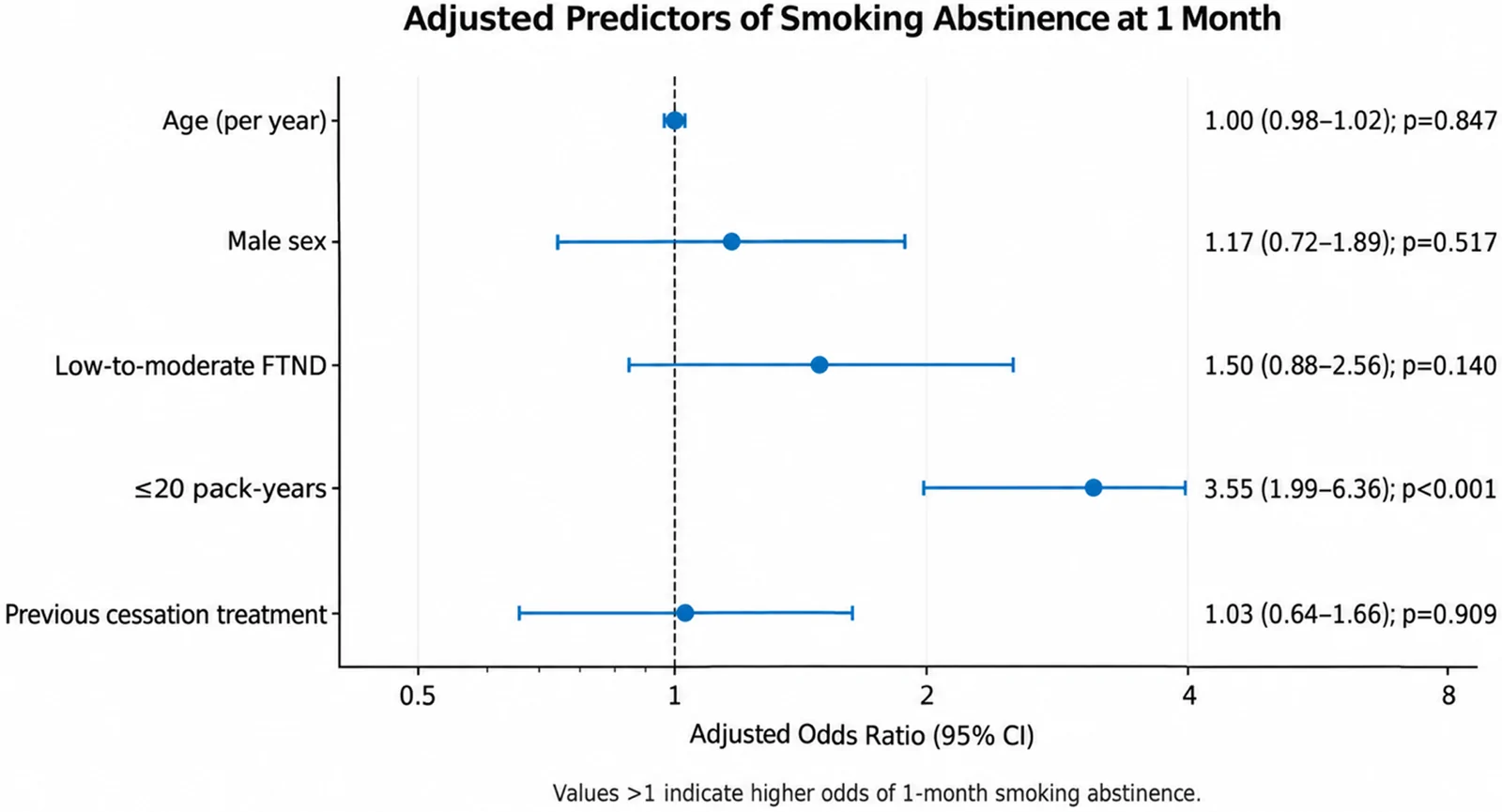
Adjusted associations with smoking abstinence at 1 month. Adjusted odds ratios and 95% confidence intervals are shown for the final model including age, sex, low-to-moderate versus high FTND, ≤20 vs >20 pack-years, and previous cessation treatment. Values >1 indicate higher odds of 1-month abstinence

Men achieved higher quit rates than women (37.1% vs 25.8%, p=0.001). Similarly, participants with low-to-moderate nicotine dependence demonstrated higher cessation rates than those with high nicotine dependence (45.3% vs 26.9%, p<0.001). Smoking exposure also showed a dose-response relationship, with patients smoking ≤20 pack-years exhibiting higher cessation rates than those with >20 pack-years (40.2% vs 26.9%, p=0.004). In addition, treatment-naïve participants achieved higher quit rates than individuals with previous smoking cessation treatment (35.3% vs 24.2%, p=0.018).

After multivariable adjustment for age, sex, nicotine dependence, cumulative smoking exposure, and previous smoking cessation treatment, smoking exposure ≤20 pack-years remained independently associated with smoking abstinence at 1 month (AOR=3.55; 95% CI: 1.99–6.36; p<0.001). In contrast, male sex (AOR=1.17; 95% CI: 0.72–1.89; p=0.517), low-to-moderate nicotine dependence (AOR=1.50; 95% CI: 0.88–2.56; p=0.140), age (AOR=1.00; 95% CI: 0.98–1.03; p=0.847), and previous smoking cessation treatment (AOR=1.03; 95% CI: 0.64–1.66; p=0.909) were not independently associated with 1-month abstinence (Figure 2).

A significant interaction was observed between nicotine dependence and smoking exposure (p=0.028), indicating that the association between cumulative smoking exposure and smoking cessation differed according to the degree of nicotine dependence. Smoking exposure demonstrated moderate discriminative ability for predicting early smoking cessation (AUC=0.66; 95% CI: 0.59–0.72), with an optimal cutoff value of 20 pack-years, corresponding to a sensitivity of 69.3% and specificity of 55.8%.

### Early treatment response and six-month smoking abstinence

Participants were stratified according to smoking status at the 1-month follow-up. Early responders (n=114; 31.3%) demonstrated substantially better long-term outcomes than early non-responders. At 6 months, smoking abstinence was maintained by 49.1% of early responders compared with 4.4% of early non-responders (p<0.001).

In multivariable analysis, early treatment response was the variable most strongly associated with sustained smoking abstinence at 6 months (AOR=9.8; 95% CI: 4.7–20.5; p<0.001). Compared with early non-responders, early responders were characterized by lower nicotine dependence, lower cumulative smoking exposure, a higher proportion of men, and lower rates of previous smoking cessation treatment.

### Predictors of failure to achieve smoking abstinence at six months

Treatment failure at 6 months occurred in 297 patients (81.6%). In multivariable logistic regression analysis, high nicotine dependence (AOR=2.14; 95% CI: 1.32–3.47; p=0.002) and higher cumulative smoking exposure (≥30 pack-years) (AOR=1.88; 95% CI: 1.12–3.15; p=0.017) were independently associated with failure to achieve smoking abstinence at 6 months (Figure 3).

**Figure 3:**
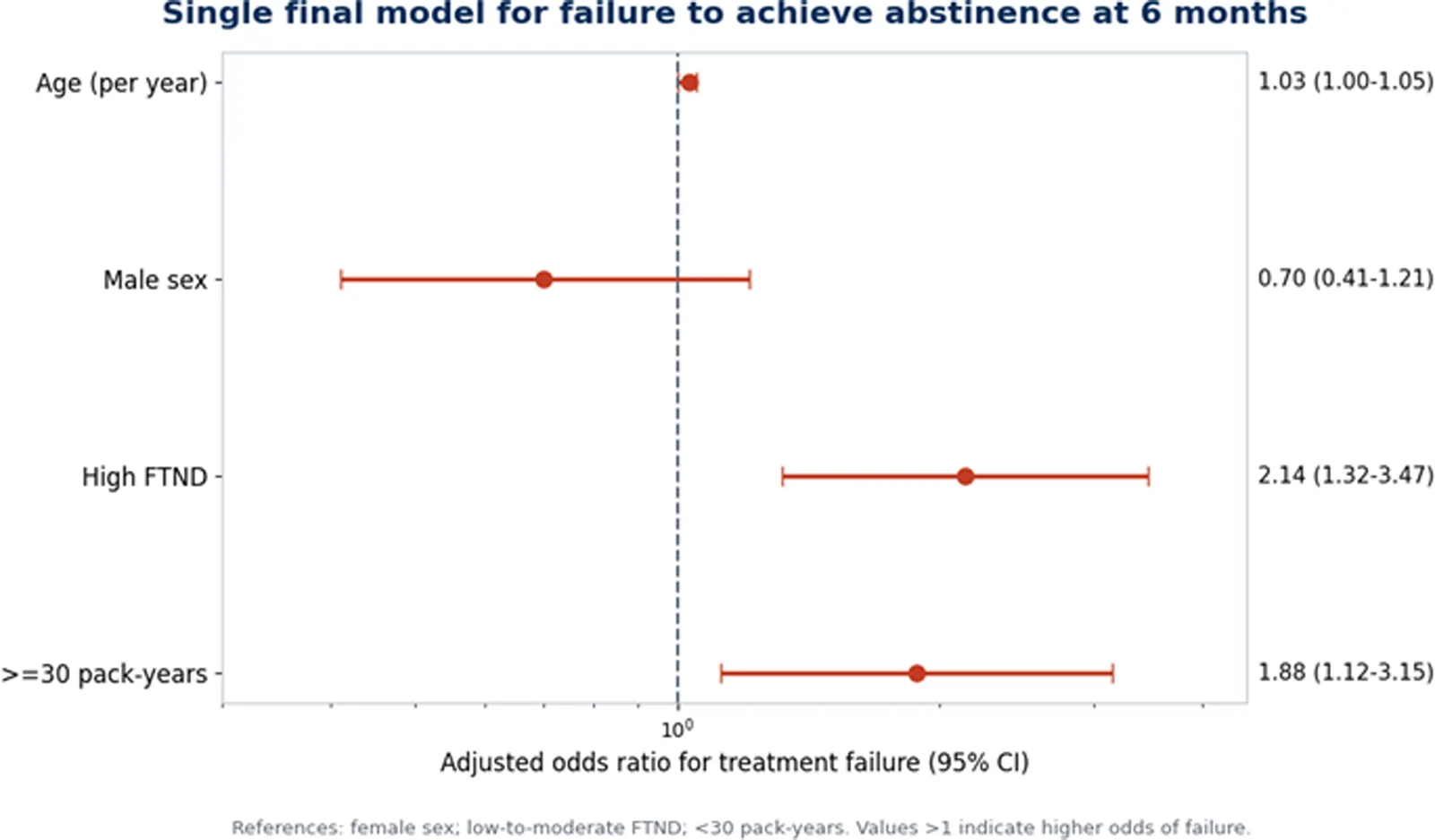
Single final multivariable logistic regression model for failure to achieve smoking abstinence at 6 months. The model used age (per year), male versus female sex, high versus low-to-moderate FTND, and smoking exposure ≥30 versus <30 pack-years. Values >1 indicate higher odds of treatment failure

Smoking exposure demonstrated moderate discriminative ability for predicting treatment failure, with an outcome-specific optimal cutoff value of approximately 30 pack-years identified by ROC analysis. A significant interaction between cumulative smoking exposure and nicotine dependence was observed, indicating that their combined effect was associated with lower smoking cessation success.

### Time-to-relapse analysis

Kaplan–Meier analysis demonstrated a median relapse-free survival of approximately 2.8–3.2 months. Participants with high nicotine dependence and smoking exposure ≥30 pack-years experienced significantly shorter relapse-free survival than their counterparts (log-rank p<0.05 for both comparisons). Early non-response was also associated with a substantially higher risk of relapse (hazard ratio=2.94; 95% CI: 2.01–4.29; p<0.001).

### Predictive model performance

A multivariable prediction model incorporating early treatment response, nicotine dependence, cumulative smoking exposure, and sex demonstrated good discriminative performance, with an AUC of 0.82 (95% CI: 0.76–0.88). When early treatment response was excluded from the model, discrimination decreased to an AUC of 0.68 (95% CI: 0.582–0.783) (Figure 4).

**Figure 4:**
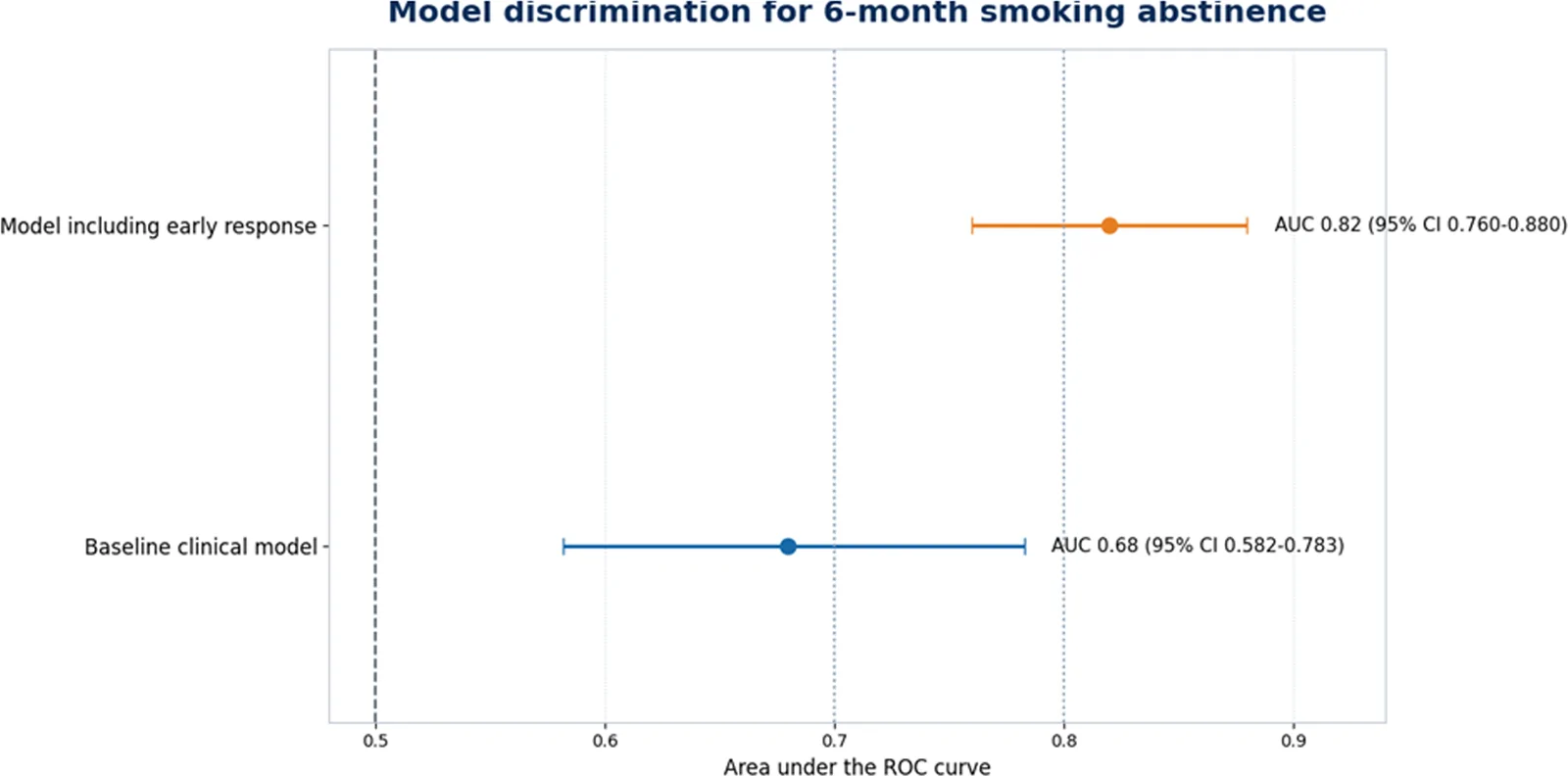
Discriminative performance of multivariable models for predicting 6-month smoking abstinence. The baseline clinical model had an AUC of 0.68 (95% CI: 0.582–0.783). Adding early treatment response increased the AUC to 0.82 (95% CI: 0.760–0.880)

## Discussion

In this real-world retrospective cohort study, a comprehensive smoking cessation program incorporating cytisine was associated with clinically meaningful smoking abstinence rates, with 31.3% of participants achieving abstinence at 1 month and 18.4% maintaining abstinence at 6 months. Beyond these overall outcomes, the principal finding of the present study was the strong association between early treatment response and sustained smoking abstinence. Participants who achieved abstinence within the first month had nearly a tenfold higher likelihood of remaining abstinent at 6 months than those who continued smoking, even after adjustment for demographic and clinical characteristics.

These findings support the concept that smoking cessation is a dynamic and time-dependent process in which early treatment response may reflect differences in biological and behavioral factors influencing treatment success. Previous studies have consistently demonstrated that early abstinence predicts long-term smoking cessation^8^. Our findings further support this concept by showing that early treatment response remained the variable most strongly associated with sustained abstinence in multivariable analyses. From a clinical perspective, assessment of smoking status during the first month may therefore provide a practical opportunity to identify individuals who could benefit from closer follow-up or treatment modification.

Consistent with previous literature, nicotine dependence severity and cumulative smoking exposure were the major contributors to smoking cessation outcomes^3,5,9-13^. In addition, we observed a significant interaction between these variables, suggesting that their combination was associated with lower smoking cessation success than either factor alone. Rather than acting independently, nicotine dependence and cumulative smoking exposure appeared to exert complementary adverse effects on treatment outcomes. These findings may help clinicians recognize patients who are more likely to require intensive behavioral support and closer follow-up during smoking cessation treatment.

The predictive analyses further demonstrated that incorporation of early treatment response substantially improved model discrimination compared with models based solely on baseline clinical characteristics. While baseline demographic and smoking-related variables showed only moderate predictive ability, adding early treatment response increased the model AUC from 0.68 to 0.82. This finding suggests that incorporating an early clinical assessment may improve risk stratification beyond baseline characteristics alone and supports the potential value of dynamic prediction models in smoking cessation practice.

Despite a cohort characterized by high nicotine dependence and substantial cumulative smoking exposure, smoking cessation outcomes observed in this study were comparable to those reported in randomized clinical trials and contemporary meta-analyses evaluating cytisine^4,5,14-18^. Comparative randomized evidence has also demonstrated clinically meaningful cessation rates with cytisine, although non-inferiority to varenicline was not established with the standard cytisine treatment regimen^17^. These findings support the effectiveness of cytisine when delivered within a structured smoking cessation program and highlight the importance of combining pharmacotherapy with behavioral interventions in routine clinical practice^15,16^.

The decline in abstinence rates between the first and sixth months reflects the well-recognized relapse pattern observed in tobacco dependence^19,20^. Although pharmacotherapy may facilitate initial smoking cessation, maintenance of long-term abstinence likely depends on sustained behavioral adaptation and continued clinical support. These findings emphasize the importance of continued follow-up after treatment initiation rather than focusing exclusively on early cessation outcomes.

Male participants demonstrated higher smoking cessation rates during the early treatment period, consistent with evidence suggesting sex differences in the ability to establish very early abstinence^21^. However, this association was attenuated after multivariable adjustment, suggesting that sex differences may be partially explained by other clinical characteristics. Similarly, education level showed a modest association with long-term abstinence, consistent with previous reports suggesting that health literacy and treatment adherence may influence smoking cessation outcomes^22,23^.

Interestingly, and in contrast to traditional assumptions, high nicotine dependence did not preclude treatment success. A substantial proportion of long-term abstinent individuals exhibited high FTND scores, suggesting that cytisine may retain efficacy even in highly dependent populations. This observation aligns with the pharmacological mechanism of partial nicotinic receptor agonists, which reduce withdrawal symptoms while attenuating nicotine reinforcement^2,24^. Although higher nicotine dependence was independently associated with failure to achieve smoking abstinence at 6 months, a considerable proportion of participants who achieved sustained abstinence also had high FTND scores. This observation suggests that successful smoking cessation remains achievable even among highly nicotine-dependent smokers and supports the use of cytisine across a broad spectrum of nicotine dependence severity.

### Strengths and limitations

The strengths of this study include its real-world design, inclusion of consecutive patients treated within a structured smoking cessation program, and comprehensive analytical approach incorporating multivariable regression, interaction analyses, receiver operating characteristic analysis, and survival analysis. The evaluation of early treatment response as a dynamic clinical variable represents an important contribution that complements traditional baseline prediction models.

Several limitations should be acknowledged. First, the retrospective single-center design limits causal inference and may reduce generalizability. Second, smoking abstinence was based on self-report without biochemical confirmation, introducing the possibility of outcome misclassification. Third, although follow-up completion was incomplete, all patients initiating cytisine treatment constituted the study cohort, and incomplete follow-up may have introduced attrition bias. Finally, potentially important determinants of smoking cessation, including psychiatric comorbidities, medication adherence, and the intensity of behavioral interventions were not available for analysis. Future multicenter prospective studies incorporating biochemical validation and external validation of prediction models are warranted. In addition, the pack-year thresholds used in the 1- and 6-month analyses were derived from outcome-specific ROC analyses within the present cohort and should therefore be considered exploratory data-driven cut-points requiring validation in independent populations.

## Conclusions

Early treatment response was strongly associated with sustained smoking abstinence and may represent a clinically useful time point for treatment reassessment. Cytisine, when combined with structured behavioral support, provides effective and scalable cessation outcomes in real-world settings. From a clinical perspective, these findings suggest that the first month after treatment initiation represents an important opportunity for reassessment. Patients who fail to achieve early abstinence may benefit from individualized treatment modification, including intensified behavioral support or alternative pharmacological strategies. However, prospective intervention studies are required to determine whether treatment adaptation based on early response improves long-term smoking cessation outcomes.
